# Total Laparoscopic Hysterectomy: Evaluation of an Evidence-Based Educational Strategy Using a Novel Simulated Suture and Knot-Tying Challenge, the “Holiotomy”

**DOI:** 10.1155/2012/592970

**Published:** 2012-02-09

**Authors:** Katherine A. O'Hanlan, Kelli R. Beingesser, Suzanne L. Dibble

**Affiliations:** ^1^Laparoscopic Institute for Gynecologic Oncology, Portola Valley, CA 94028-8015, USA; ^2^Gynecologic Oncology Associates, 4370 Alpine Road, Suite 104, Portola Valley, CA 94028, USA; ^3^Fresno Women's Medical Group, Fresno, CA 93720, USA; ^4^School of Nursing, University of California at San Francisco, San Francisco, CA 94143, USA

## Abstract

*Objective*. The purpose of this study was to evaluate perceptions of skills and practice patterns of gynecologists attending a course on total laparoscopic hysterectomy (TLH). This course employed extensive use of pelvic trainer boxes to accomplish the Holiotomy Challenge. The “Holiotomy Challenge” entailed suturing two plastic pieces with six figure-of-N sutures tied with four square knots each. *Methods*. A survey was administered before the course and 3 months later. Data were analyzed by paired *t*-tests, McNemar's Chi Squares, and ANCOVAs with significance set *P* < .05. *Results*. At baseline, 216 surgeons and at 3 months 102 surgeons returned the survey. Surgeons' self-perceptions of their skills significantly increased from 6.24 to 7.28. Their reports of their surgical practice at home revealed significantly increased rates of minimally invasive procedures, from 42% to 54%. Significantly more surgeons reported having the ability to close the vagina, or a small cystotomy or enterotomy. Participation in the cadaver lab and presence of their practice partner did not impact these rates. *Conclusions*. A comprehensive course employing laparoscopic surgical simulation focused on basic surgical skills essential to TLH has a positive impact on attendees' self-rated skill level and rate of laparoscopic approaches. Many had begun performing TLH after the course.

## 1. Introduction

Total laparoscopic hysterectomy has been shown to be a safe method of hysterectomy with minimal complications [1], yet only 12% of hysterectomies are performed by this route, with 22% by vaginal approach and 66% still being performed by laparotomy [2]. Surgeons have been encouraged to employ vaginal and laparoscopic routes for hysterectomy, but concerns exist about how to increase laparoscopic suturing skills without elevating risk to patients [3]. Currently available educational methods include broadly focused annual continuing medical education courses, mail-order instructional videos, informal mentoring, suture skills, and, more recently, comprehensive courses focused entirely on total laparoscopic hysterectomy and its component skills. Such courses combine videos, slide lectures, and precepted and laparoscopic practice simulation trainers all focused on the specific steps to perform minimally invasive surgery [4]. The impact of such a comprehensive course on the gynecologic surgeon's self-perceived skill level and practice patterns has not been established.

Since 2004, a course focused on total laparoscopic hysterectomy (TLH) has been jointly sponsored by the American College of Obstetricians and Gynecologists for continuing medical education of gynecologic surgeons. This course extensively employs surgical simulators to train surgeons in laparoscopic suturing and knot tying. A simulation for suturing was developed to require that six “figure-of-N” sutures be placed through twelve dots and required four square knots to close. This “Holiotomy” was completed by 88% of surgeons. It is hypothesized that a comprehensive course employing simulators would improve participant's self-perceived laparoscopic skill levels. It was further hypothesized that after three months these changes would manifest with more TLHs and other minimally invasive surgeries being reported in their practice pattern.

## 2. Methods

Investigational Review Board approval of the survey protocol was obtained through Sequoia Hospital in Redwood City, California. The survey (see 2009 LIGO COURSE ATTENDEE QUESTIONAIRE) was distributed to all physician attendees at the Laparoscopic Institute for Gynecologic Oncology 4th annual course on Total Laparoscopic Hysterectomy. It was collected before the first morning break. Each questionnaire was numbered and stapled to a sealed, stamped envelope containing a similarly numbered questionnaire with a self-addressed stamped envelope for return. The attendees addressed the outer envelopes to themselves and handed these in with the completed precourse survey. The hand-addressed envelopes containing the second survey and a stamped return envelope were mailed to the course participants 90 days after completion of the course.

2009 LIGO COURSE ATTENDEE QUESTIONAIRE

Age_  
Gender_
Year of fellowship completion_N/A
Practice type:
  Private Practice
  University Practice
  Resident MD
  Fellow MD
How would you rate your own overall performance of laparoscopic surgical skills?
  1 2 3 4 5 6 7 8 9 10
  (10 is performing nearly all abdominal procedures laparoscopically and 1 is only performing laparoscopic tubal ligations)
Do you have a practice partner with whom you perform most laparoscopic procedures?
  Yes
  No
How would you rate your partner's laparoscopic skills?
  1 2 3 4 5 6 7 8 9 10
  (Use same scale as above)
Did you attend this 2009 LIGO Course with that practice partner?
  Yes
  No
How would you rate your urogynecologic skills?
  1 2 3 4 5 6 7 8 9 10
  (10 is rarely referring to patients for urological procedures and 1 is referring to all patients)
Do you currently have advanced laparoscopic privileges at you hospital?
  Yes
  No
  I assume I do
Does your hospital have a formal process to add new surgical procedures to your repertoire?
  Yes
  No
If so, how many proctored cases are required?—
During the past 2 months, how many of the following have you performed? (Exclude vacation weeks)
  TOTAL ABDOMINAL HYSTERECTOMY_
  TOTAL VAGINAL HYSTERECTOMY_
  LAPAROSCOPIC ASSISTED VAGINAL HYSTERECTOMY_
  TOTAL LAPAROSCOPIC HYSTERECTOMY_
  LAPAROSCOPIC SUPRACERVICAL HYSTERECTOMY_
  ENDOMETRIAL ABLATION_
  LAPAROSCOPIC SACROCOLPOPEXY_
  SUBURETHRAL VAGINAL SLING_
I am able to perform cystoscopy during some surgeries in my practice.
  Yes
  No
I am able to laparoscopically close the vagina after hysterectomy.
  Yes
  No
I am able to laparoscopically close a 1cm cystotomy in the dome of the bladder.
  Yes
  No
I am able to laparoscopically close a 1 cm enterotomy in the sigmoid colon.
  Yes
  No
Did you attend the 2009 LIGO cadaver lab?*
  Yes
  No
*These questions were not in the second questionnaire.

### 2.1. Curriculum

This course employed multiple techniques for learning. Didactic lectures using referenced slide presentations were used to teach electrosurgical safety, laparoscopic surgical anatomy, avoidance and management of intestinal and urological complications, and coding for all procedures mentioned. Richly edited videos of TLH and advanced pelvic surgeries comprised most of the 26 hours of the three-day course. Four surgeons established in their own TLH technique focused on common obstacles in performing TLH: the parametrial dissection and closure of the vaginotomy. Faculty videos demonstrated procedures typically performed concomitant with TLH, including uterosacral ligament plication, endometriosis resection, ureterolysis, enterocele repair, burch procedure, cystoscopy, and appendectomy. Advanced support and gynecologic surgeries such as myomectomy, colposuspension, vaginal hysterectomy, and other mesh procedures were shown. Three faculty members showed detailed videos of suturing and knot tying, with live plenary session demonstration of suture techniques followed immediately by faculty precepted sessions of simulated laparoscopic suturing and knot-tying.

**Figure 1:**
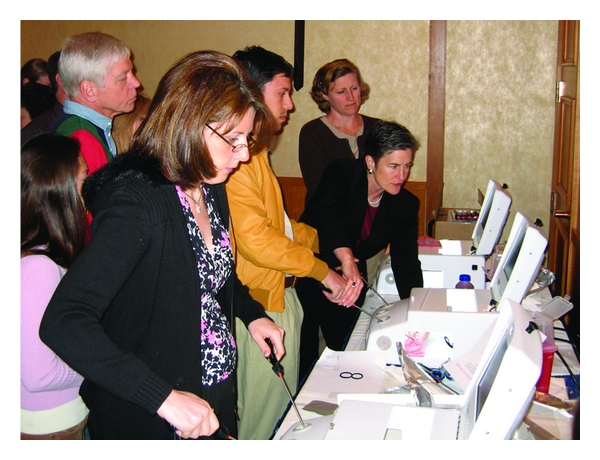
Surgeons work with supervision to complete their Holiotomy challenges using laparoscopic simulator trainer boxes.

**Figure 2:**
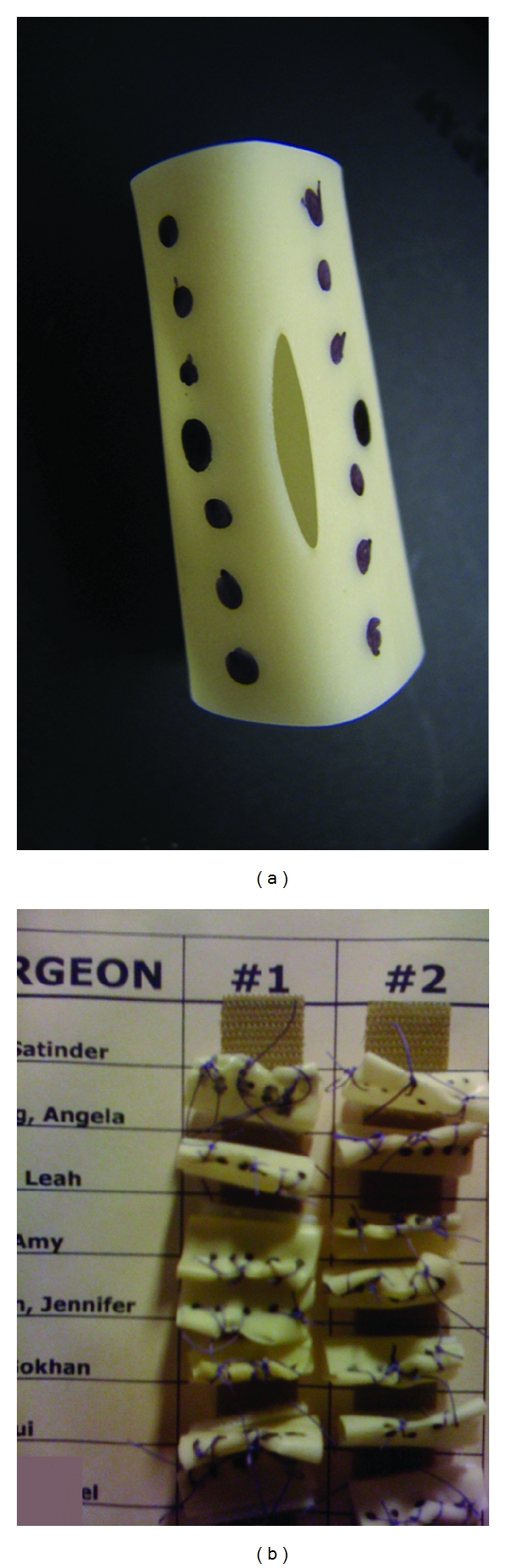
(a) This “Holiotomy” is marked with dots on each side, which surgeons must suture through in placing three “figure of N's” and then tie each with four square knots. Thus, twenty-four sutures are passed through a dot, and at least twenty-four knots are tied. (b) Close-up of completed holiotomies on the board.

**Figure 3:**
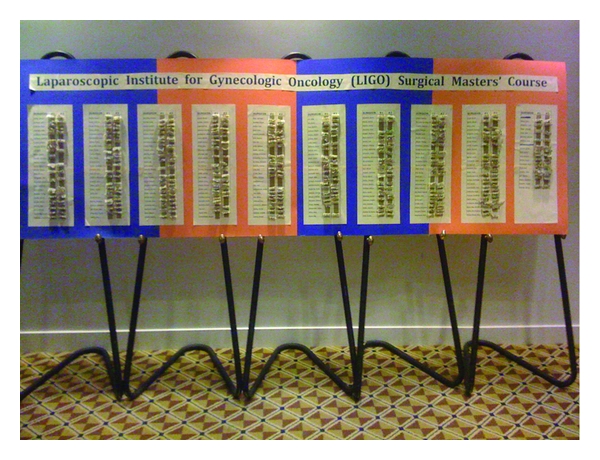
The first Holiotomy board attested to completion of the Holiotomy challenge, and revealed participation and completion by 88% of the 225 attendees.

The twenty-two faculty members were assigned to precept four attendees at each of four 45-minute sessions at the pelvic trainers. Attendees were precepted in both suturing and knot tying, and to complete the “Holiotomy challenge.” (Figures 1 and 2). A “Holiotomy” is the name used in the course for a 4 cm segment of a penrose drain, attached by Velcro to the floor of the pelvic trainer box suture area. Six dots were placed on each side of a 2 cm hole cut into the top side. The challenge was to place three “figure of N” sutures, precisely through each of the dots, and then tie with at least four throws of a square knot, usually many more. Surgeons were asked to hand in at least two holiotomies, which meant that they had placed over 24 sutures through a small dot and tied over 24 knots. The holiotomies were then attached by their Velcro base near the surgeons name on a prominently placed poster board to acknowledge the accomplishment and enhance esprit de corps (Figure 3). The pelvic trainers were unassigned and available to all attendees at all other times during the course to enable as much practice time as they chose.

Finally, an optional 4-hour cadaver dissection session with four surgeons and one faculty to each specimen was available to 120 attendees. General gynecologic surgeons first performed TLH, then other advanced laparoscopic procedures such as ureterolysis, appendectomy, burch colposuspension, and uterosacral ligament colposuspension, while gynecologic oncologist attendees performed retroperitoneal aortic and pelvic lymphadenectomy and radical hysterectomy. This optional segment was accompanied by four lectures on challenging hysterectomies such as for the obese, the elderly, or those with adhesions or massive fibroids.

### 2.2. Data Management

Data were entered into Excel, cleaned, and then uploaded into SPSS (Version 17) for analyses. Sample descriptive statistics were generated and more complex statistics were calculated based upon the research questions. Because we had paired data, we were able to use statistics that are specific for this type of data including paired *t*-tests and McNemar's Chi Squares. ANCOVAs were also performed [5]. Significance was preset at *P* < .05.

## 3. Results

Of the 216 participants in the course, 102 returned their second evaluation forms for a response rate of 47%. The typical participant was female (62%), did not complete a fellowship (90%), and had an average age of 44.7 years. There were no significant differences in age or gender in the responders versus the nonresponders. Among all course participants, 4% were residents, 77% were in private practice, and 18% were in university practice.

Attendees were asked how many of each kind of surgeries they recalled performing in the prior two months: total abdominal hysterectomy (TAH), total vaginal hysterectomy (TVH), laparoscopic assisted vaginal hysterectomy (LAVH), total laparoscopic hysterectomy (TLH), laparoscopic supracervical hysterectomy (LSH), endometrial ablation (EA), laparoscopic sacrocolpopexy (LSCP), and suburethral vaginal sling (SVS).  Table 1 contains the numbers of various surgeries by type before and after the course with asterisks to identify the minimally invasive procedures taught in the course. The average total number of reported surgeries performed over a two-month period before the course was 14.05 (SD = 8.2), which did not change significantly after the course (*P* = .498). However, types of procedures did change significantly (*P* = .001) after the course. The number of minimally invasive surgeries (TVH, LAVH, TLH, and LSCP) increased from 6.28 to 7.55 over a two-month period, as did the percent of minimally invasive surgeries as a portion of the total (42% to 54%, *P* < .001).

The participants rated their own initial laparoscopic skill on a scale from 1 to 10 with 10 being the best, at a mean of 6.24 ± 1.5 before the course, and later rated themselves a mean of 7.28 ± 1.4, a significant improvement (*t* = −9.17, *P* < .001). The participants also rated their own initial urogynecologic surgical skill on a scale from 1 to 10 with 10 being the best, with a mean of 4.52 ± 2.5. The postcourse mean rating of 4.93 ± 2.6 (*t* = −2.49, *P* < .014) reflected a significant improvement.

Since the course focused very specifically on TLH skills, the final survey questions asked surgeon attendees before and three months later just how comfortable they were performing four of the major portions of TLH and related procedures that were taught at the course.  Table 2 contains the types of skills reportedly performed over a typical two-month period both before and after the course. Significantly more surgeons felt that they could comfortably suture close the vagina, perform laparoscopic cystoscopy, and close a small cystotomy or enterotomy after their training compared to before the training.

This course had an optional cadaver lab, and 50% of the participants took advantage of this opportunity. Controlling for precourse self-rated laparoscopy skill, participation in the cadaver lab did not make a significant difference in the self-rated skill of the participant (*P* = .340) three months after course. Controlling for precourse self-rated urogynecologic skills, participation in the cadaver lab did not make a significant difference in the self-rated urogynecologic skills of the participant (*P* = .250) three months after course. In addition once precourse data were controlled, participation in the cadaver lab did not make a significant increment in the number (*P* = .689) or percent of minimal invasive surgeries (*P* = .858) three months after course.

Most (*n* = 127, 59%) of the participants reported having a practice partner when they performed most laparoscopic procedures and 58% (*n* = 73) of these partners were also taking the course. Controlling for precourse self-rated laparoscopy skill, having their practice partner at the course did not make a significant difference in the self-rated skill of the participant (*P* = .414) three months after course. Controlling for precourse self-rated urogynecologic skills, having their practice partner at the course did not make a significant difference in the self-rated urogynecologic skills of the participant (*P* = .084) three months after course. In addition once precourse data were controlled, having their practice partner at the course did not make a significant difference in the number (*P* = .469) or percent of minimal invasive surgeries (*P* = .305) three months after course.

## 4. Discussion

Practicing gynecologists need an effective means for learning new skills and procedures in laparoscopic surgery, including hysterectomy. It has been shown that a focused hands-on course can produce quantifiable improvements in laparoscopic skills [6–8]. Surgical simulation using video trainer boxes has been demonstrated to lead to greater dexterity and efficiency, as well as comfort performing complex laparoscopic procedures [9]. Residents trained on laparoscopic surgery simulators showed improvement in procedural performance that translated to improved efficacy in the operating room [10]. Surgeons trained in courses offering skills-based lectures, surgical video analysis, precepted pelvic trainer performance, and precepted cadaver laboratory experienced significant expansion of their minimal invasive surgical practice, including suturing [7, 10]. It has been shown that focused courses on laparoscopic ventral herniorrhaphy and splenectomy can increase the number of minimally invasive procedures that general surgeons employ in their armamentariom [11, 12], but such evidence has not been reported for gynecologic surgeons performing hysterectomy.

All course attendees were exhorted to complete the Holiotomy challenges after an explanation of their evidence-basis, which allowed surgeons to develop their psychomotor and manual dexterity skills in a low-stress environment, enhancing muscle memory, and proven to translate into operating room skills [13]. While the “Holiotomy challenge” has not been validated, per se, it is based on published evidence that 5–7 repetitions of intracorporeal knot-tying in trainer boxes effectively enhanced efficiency and translated well into operating room skills [14–16]. The Holiotomies and the trainer boxes simulated the most difficult tasks during a total laparoscopic hysterectomy: the parametrial dissection and the closure of the vaginotomy. The questions and tabulated answers in Table 2 focus on the most difficult tasks taught in the course, which required the most dexterity and skill to perfrom.

It has been shown that surgeons who attended a laparoscopic surgical training course alone or who routinely performed laparoscopic surgery with random surgical assistants were almost five times more likely to have had a complication than their counterparts who attended the course with a partner or who operated consistently with the same assistant [17]. We thus encouraged attendees to bring their surgical partner, theorizing that self-rated skills would rise more if learning and subsequent practice were undertaken with a similarly trained partner. However, only a trend was observed (*P* = .084) that surgeons with practice partners attending the course developed higher postcourse urogynecologic skills. Our survey was not adequately constructed to match the practice pairs (*n* = 37), so this comparison cannot be adequately made at this time. Future surveys will pair the partners so that this concept can be further explored.

This study design is susceptible to bias and error and, as such, these results cannot conclude that the educational opportunity meaningfully changed practice patterns. Participation in the 3-month follow-up questionnaire and even one's self-perceived skill levels assessed on a Likert-scale three months separate in time are subject to bias. Laparoscopic surgeons have been shown to rate their skills higher than objective testing confirms [18], and having taken the course may cause respondents to self-rate more highly, resulting in a false but statistically significant increase. It is possible that the surgeon attendees who participated in the 3-month survey were more confident, more successful, or possibly the opposite, than those who declined, even though they were not different with regard to baseline characteristics.

The entirely subjective nature of the numerical data, relying on recall of surgeries performed and estimation of two-months practice pattern, is also subject to error. Laparoscopic surgeons may also perform more minimally invasive surgeries after a course, not as a result of learning from a course, but as a function of having a certificate obtained from attendance at the course. Perceptions of one's past two months' typical practice patterns may still vary, especially by recency of vacation or holiday time. Objective measurements of laparoscopic skill and dexterity have been performed [19] and could be added to future course surveys to lend validity to the course material and teaching modalities. It would also be useful to know which of the attendees completed their Holiotomy challenges, and whether that affected their future ratings.

The survey response rate of 47% from a single mailing is actually quite good [20]. Other laparoscopic course follow-up surveys reported a postcourse response rate of 79% [7, 17]; however they used multiple and repeated modalities to obtain this rate whereas we could not, given the original plan for a single anonymous mailing to all. Future questionnaires for this course material will employ an established internet-based survey application for easier obtaining and collation of response data and will employ repeated requests to participate. This should increase likelihood of follow-up participation and enhance accuracy of results.

## 5. Conclusion

Practicing surgeons need an effective means for learning suture and knot tying skills and procedures in advanced gynecologic laparoscopy. It is possible that the “Holiotomy” facilitated clinical uptake of laparoscopic skills and enhanced the effectiveness of this comprehensive course.

## Figures and Tables

**Table 1:** Numbers of gynecological surgeries (*n* = 99).

Type of surgery	2 mo. before course	Months—2 to 3 after the course	Statistic (paired *t*)	Significance
Total laparoscopic hysterectomy**	0.98	1.99	*t* = −5.66	*P* < .001
Total vaginal hysterectomy**	1.92	2.13	*t* = −1.10	*P* = .275
Laparoscopic assisted vaginal hysterectomy**	1.72	1.67	*t* = 0.21	*P* = .835
Laparoscopic sacrocolpopexy**	0.12	0.13	*t* = −0.26	*P* = .798
Total abdominal hysterectomy	2.49	2.03	*t* = 1.72	*P* = .089
Laparoscopic supracervical hysterectomy	1.21	0.79	*t* = 2.84	*P* = .005
Endometrial ablation	3.99	3.15	*t* = 2.80	*P* = .006
Suburethral vaginal sling	1.71	1.7	*t* = .061	*P* = .951

** Minimally invasive procedures taught in the course.

**Table 2:** Skill changes*.

Skill	% Yes before course	% Yes after course	Significance
Perform cystoscopy during some surgeries in my practice	74	84	*P* = .039
Laparoscopically close the vagina after hysterectomy	33	56	*P* < .001
Laparoscopically close a 1 cm cystotomy in the dome of the bladder.	22	52	*P* < .001
Laparoscopically close a 1 cm enterotomy in the sigmoid colon	6	23	*P* = .001

*McNemar's Chi-Square.

## References

[1] O’Hanlan KA, Dibble SL, Garnier AC, Reuland ML (2007). Total laparoscopic hysterectomy: technique and complications of 830 cases. *Journal of the Society of Laparoendoscopic Surgeons*.

[2] Wu JM, Wechter ME, Geller EJ, Nguyen TV, Visco AG (2007). Hysterectomy rates in the United States, 2003. *Obstetrics and Gynecology*.

[3] Walsh CA, Walsh SR, Tang TY, Slack M (2009). Total abdominal hysterectomy versus total laparoscopic hysterectomy for benign disease: a meta-analysis. *European Journal of Obstetrics Gynecology and Reproductive Biology*.

[4] Birch DW, Sample C, Gupta R (2007). The impact of a comprehensive course in advanced minimal access surgery on surgeon practice. *The Canadian Journal of Surgery*.

[5] De Muth JE (2009). Overview of biostatistics used in clinical research. *The American Journal of Health-System Pharmacy*.

[6] Torkington J, Smith SG, Rees B, Darzi A (2001). The role of the basic surgical skills course in the acquisition and retention of laparoscopic skill. *Surgical Endoscopy*.

[7] Pareek G, Hedican SP, Bishoff JT, Shichman SJ, Wolf JS, Nakada SY (2005). Survey from skills-based hands on learning courses demonstrates increased laparoscopic caseload and clinical laparoscopic suturing. *Urology*.

[8] Melvin WS, Johnson JA, Ellison EC (1996). Laparoscopic skills enhancement. *The American Journal of Surgery*.

[9] Gettman MT, Pereira CW, Lipsky K (2009). Use of high fidelity operating room simulation to assess and teach communication, teamwork and laparoscopic skills: initial experience. *Journal of Urology*.

[10] Lehmann KS, Ritz JP, Maass H (2005). A prospective randomized study to test the transfer of basic psychomotor skills from virtual reality to physical reality in a comparable training setting. *Annals of Surgery*.

[11] Heniford BT, Matthews BD, Box EA (2002). Optimal teaching environment for laparoscopic ventral herniorrhaphy. *Hernia*.

[12] Heniford BT, Backus CL, Matthews BD, Greene FL, Teel WB, Sing RF (2001). Optimal teaching environment for laparoscopic splenectomy. *The American Journal of Surgery*.

[13] Kanumuri P, Ganai S, Wohaibi EM, Bush RW, Grow DR, Seymour NE (2008). Virtual reality and computer-enhanced training devices equally improve laparoscopic surgical skill in novices. *Journal of the Society of Laparoendoscopic Surgeons*.

[14] Van Sickle KR, Ritter EM, Baghai M (2008). Prospective, randomized, double-blind trial of curriculum-based training for intracorporeal suturing and knot tying. *Journal of the American College of Surgeons*.

[15] Goff BA, Lentz GM, Lee D, Houmard B, Mandel LS (2000). Development of an objective structured assessment of technical skills for obstetric and gynecology residents. *Obstetrics and Gynecology*.

[16] Stelzer MK, Abdel MP, Sloan MP, Gould JC (2009). Dry lab practice leads to improved laparoscopic performance in the operating room. *Journal of Surgical Research*.

[17] See WA, Cooper CS, Fisher RJ (1993). Predictors of laparoscopic complications after formal training in laparoscopic surgery. *Journal of the American Medical Association*.

[18] Sidhu RS, Vikis E, Cheifetz R, Phang T (2006). Self-assessment during a 2-day laparoscopic colectomy course: can surgeons judge how well they are learning new skills?. *The American Journal of Surgery*.

[19] Hance J, Aggarwal R, Moorthy K, Munz Y, Undre S, Darzi A (2005). Assessment of psychomotor skills acquisition during laparoscopic cholecystectomy courses. *The American Journal of Surgery*.

[20] VanGeest JB, Johnson TP, Welch VL (2007). Methodologies for improving response rates in surveys of physicians: a systematic review. *Evaluation and the Health Professions*.

